# “Ideal employees” and “good wives and mothers”: Influence mechanism of bi-directional work–family conflict on job satisfaction of female university teachers in China

**DOI:** 10.3389/fpsyg.2023.1166509

**Published:** 2023-04-11

**Authors:** Qiaolan Su, Man Jiang

**Affiliations:** ^1^School of Tourism and Sport Health, Hezhou University, Hezhou, China; ^2^Chinese International College, Dhurakij Pundit University, Bangkok, Thailand

**Keywords:** work family conflict, job satisfaction, job burnout, perceived organizational support, female teacher

## Abstract

Work and family are two important areas in people’s life, and the relationship between them will have an important impact on the attitudes and behaviors of employees in an organization. In the context of Chinese culture, the organization hopes to have an ideal employee, and the family hopes to have a good wife and mother. Based on the resource conservation theory, this paper examines the relationship between bi-directional work–family conflict and job burnout, perceived organizational support and job satisfaction by using latent variable path analysis method with 527 Chinese female university teachers as subjects. The results showed that: (1) Work–family conflict, family–work conflict and job burnout could negatively predict job satisfaction, while perceived organizational support could positively predict job satisfaction, (2) Job burnout played a partial mediating role in the process of work–family conflict and family–work conflict affecting job satisfaction, and the effect values were − 0.220 and − 0.168, and (3) Perceived organizational support played a moderating role in the first half of the mediating effect of “work–family conflict → job burnout → job satisfaction” and “family–work conflict → job burnout → job satisfaction,” and the moderating mediating index was 0.015 and 0.010. The study contributes to a better understanding of the relationships among bi-directional work–family conflict, job burnout, perceived organizational support, and job satisfaction among female university teachers. Our findings highlight potential avenues for interventions by university administrators in the context of Chinese culture aimed at balancing work and family and improving job satisfaction among female university teachers.

## 1. Introduction

With the development of a series of institutional environments, such as the globalization of higher education, academic capitalism and the ranking of world universities, the roles of university teachers have become more diverse, shouldering multiple responsibilities such as scientific research, teaching and social public services (Luo and Shen, 2017). However, in the context of China, the institutional norms of “ideal employees” and the social norms of “good wives and mothers” both put forward expectations for female teachers in universities, but it is often difficult to balance the two of them (Blair-Loy, 2009). Therefore, female college teachers need to spend a lot of time and energy, endure great pressure and try to balance the conflict between family and work to complete their work tasks (Wilton and Ross, 2017). Bi-directional work–family conflict is a special form of two-way role conflict from work and family life, in which some role pressures are incompatible. From the perspective of orientation, work–family conflict has two types of conflict structures: work–family conflict (WFC) and family–work conflict (FWC) (Frone et al., 1992). A large number of studies have shown that work–family conflict has a significant impact on the work and life of employees. The work-related outcomes mainly include job satisfaction, organizational commitment, turnover intention, absenteeism, job performance, career satisfaction and career achievement. The non-work-related outcomes mainly include life satisfaction, marital satisfaction and family satisfaction (Grandey and Cropanzano, 1999; Allen et al., 2000).

The job satisfaction of university teachers is very important, which is not only related to their work, study and life, But also affect teachers’ job involvement, job performance, loyalty, organizational commitment, OCB and intention to leave (Gao, 2021). Although many scholars have paid attention to university teachers’ job satisfaction and its formation mechanism, there are still some shortcomings in previous studies on the relationship between work–family conflict and job satisfaction. Most of the existing studies are carried out in the context of western culture, on the other hand, there are also inconsistent research results (Allen et al., 2012; Powell and Eddleston, 2013). People’s understanding of the relationship between work and family will be influenced by values, social class, social behavior norms and so on (Lin et al., 2013). In the context of Chinese culture, there is a widespread and strong social norm of work priority, a stronger tolerance of work interfering with family, and it is a traditional virtue to work hard at the expense of personal family (Lin et al., 2013; Gao, 2021).

Previous studies have found that the relationship between work–family conflict and job burnout is more obvious (Lin et al., 2013; Li et al., 2021). Role ambiguity, overload or conflict at work will increase the job stress of employees, which will lead to job burnout. This burnout and stress can have a negative impact on job satisfaction. Therefore, higher levels of work–family conflict will directly lead to greater levels of job burnout, which in turn will reduce employee job satisfaction (Zhang et al., 2011). Perceived organizational support plays a role in determining employee attitudes and behaviors. The extent to which employees feel that the organization values their work contributions and also cares about their well-being is the basis of perceived organizational support (Eisenberger et al., 2001). High organizational support is an important work resource from outside the individual, which promotes a higher level of work engagement and compensates for the negative impact of excessive work demands on individual health and well-being (de Jonge et al., 2008). Others found that organizational support has a moderating effect on the relationship between work–family conflict and job burnout (Lee and Peccei, 2007; Gao, 2021). Therefore, perceived organizational support can moderate the relationship between work–family conflict and job burnout.

Based on this, this study will further explore the relationship between work–family conflict and job satisfaction of female university teachers in China and its internal mechanism (the mediating role of job burnout and the moderating role of perceived organizational support), in order to provide reference suggestions and measures for improving job satisfaction of female university teachers.

## 2. Research hypothesis

### 2.1. Theoretical deduction

According to the theory of resource conservation, People always strive to obtain and protect resources that are valuable to them (Hobfoll, 2001). At work, individuals feel pressure from work when they feel threatened by the loss of their valuable resources, or that resources are not effectively replenished after they are invested. As “engineers of human soul,” female university teachers undertake extremely heavy teaching and scientific research work all the year round, and the high intensity of the work takes up too much time of female university teachers, thus affecting the fulfillment of their family roles and obligations which will easily lead to bi-directional work–family conflict (Zhang et al., 2011). When dealing with the work–family conflict faced by female university teachers, they need to mobilize personal resources and self-control resources to deal with the conflict. Female university teachers need to constantly carry out self-intervention to cope with the problems brought about by bi-directional work–family conflict (Hobfoll et al., 2018). If individuals frequently organize or self-intervene in order to improve their work, it will eventually lead to negative emotions (Wanous et al., 1997). Frequent self-intervention will consume a lot of psychological resources of female university teachers, resulting in a sense of emotional exhaustion and a decrease in self-efficacy, and eventually lead to job burnout (Li et al., 2021). In this process, female university teachers are under the pressure from both family and work for a long time, and their own resources are constantly consumed, which leads to the decrease of job satisfaction.

### 2.2. Bi-directional work–family conflict and job satisfaction

Studies have shown that work–family conflict can negatively predict the degree of job satisfaction. When individuals experience work–family conflict, the resources of family roles that are important to individuals are occupied by work roles, resulting in the loss of family role resources (Hobfoll et al., 2018). The loss of family role resources breaks the balance of resources, and at the same time leads to the failure of family responsibilities to be fulfilled normally, which leads to individual family pressure (Wilton and Ross, 2017). And this pressure will be transmitted to the work through the spillover effect, in order to reduce work pressure and relieve tension, individuals need to consume a lot of work resources to achieve a state of psychological balance (Hobfoll, 2001). When employees experience conflicts, they will use their limited resources to solve the problems they face, and in the process of resource consumption, it will make employees unable to have more resources to work, thus reducing the job satisfaction of employees (Greenhaus and Powell, 2006). Based on this, this study proposes the following hypotheses:

*H1*: Work-family conflict negatively predicts job satisfaction.

*H2*: Family-work conflict negatively predicts job satisfaction.

### 2.3. Mediating effect of job burnout

Work–family conflict may affect individual job satisfaction through specific ways. Job burnout refers to the emotional, attitudinal and behavioral exhaustion reaction of individuals under the pressure of long-term work (Maslach and Leiter, 2016). Job burnout includes serious psychological and/or physical problems resulting from chronic stress and/or work frustration (Maslach and Leiter, 2016). The Maslach Burnout Inventory (MBI) classifies burnout into three components: emotional exhaustion, depersonalization, and reduced personal accomplishment. A large number of studies have shown that job burnout is closely related to job satisfaction, and good job attitude and behavior can predict higher job performance (Khamisa et al., 2015) There is a significant negative correlation between job burnout and job satisfaction. When an employee is more stressed, the more likely he is to burn out, which ultimately leads to lower job satisfaction and the greater the likelihood of burnout (Madigan and Kim, 2021). Job satisfaction has been shown to be negatively affected by emotional exhaustion, cynicism, and lack of job efficacy. Based on this, this study proposes the following hypotheses:

*H3*: Job burnout plays a mediating role in the negative impact of work-family conflict on job satisfaction.

*H4*: Job burnout plays a mediating role in the negative impact of family-work conflict on job satisfaction.

### 2.4. Moderating effect of perceived organizational support

In addition, this paper proposes that the indirect process of work–family conflict affecting job satisfaction may be conditional, that is, it will be regulated by certain factors. According to organizational support theory, when employers are perceived to value and support employees, employees believe that their organizations value and care about their well-being (Eisenberger et al., 1986). Therefore, POS can be used as an indicator of organizational goodwill. Supportive and developmental supervisory behaviors, such as providing useful feedback or discussing specific challenges in the workplace, will encourage employees to reshape their work boundaries (Wanasida et al., 2021). According to this logic, if the position is higher, there will be more job opportunities. In addition, POS provides emotional support, positive self-esteem, recognition and a sense of belonging and all of these increase work engagement (Lee and Peccei, 2007; Zacher and Winter, 2011). Applying the principle of reciprocity, employees with high POS feel obligated to respond to the organization with a positive work attitude and beneficial organizational behavior (Rhoades and Eisenberger, 2002). Therefore, high POS is negatively related to job burnout and positively related to job satisfaction (Riggle et al., 2009). Based on this, perceived organizational support may affect the effect of work–family conflict on job satisfaction, that is, compared with employees with low perceived support, the effect of work–family conflict on job satisfaction is stronger in employees with high perceived organizational support. Based on this, this study proposes the following hypotheses:

*H5a*: Perceived organizational support moderates the effect of work-family conflict on job satisfaction.

*H5b*: Perceived organizational support moderates the effect of family-work conflict on job satisfaction.

*H5c*: Perceived organizational support moderates the effect of work-family conflict on job burnout.

*H5d*: Perceived organizational support has a moderating effect on the impact of family-work conflict on job burnout.

**Figure 1:**
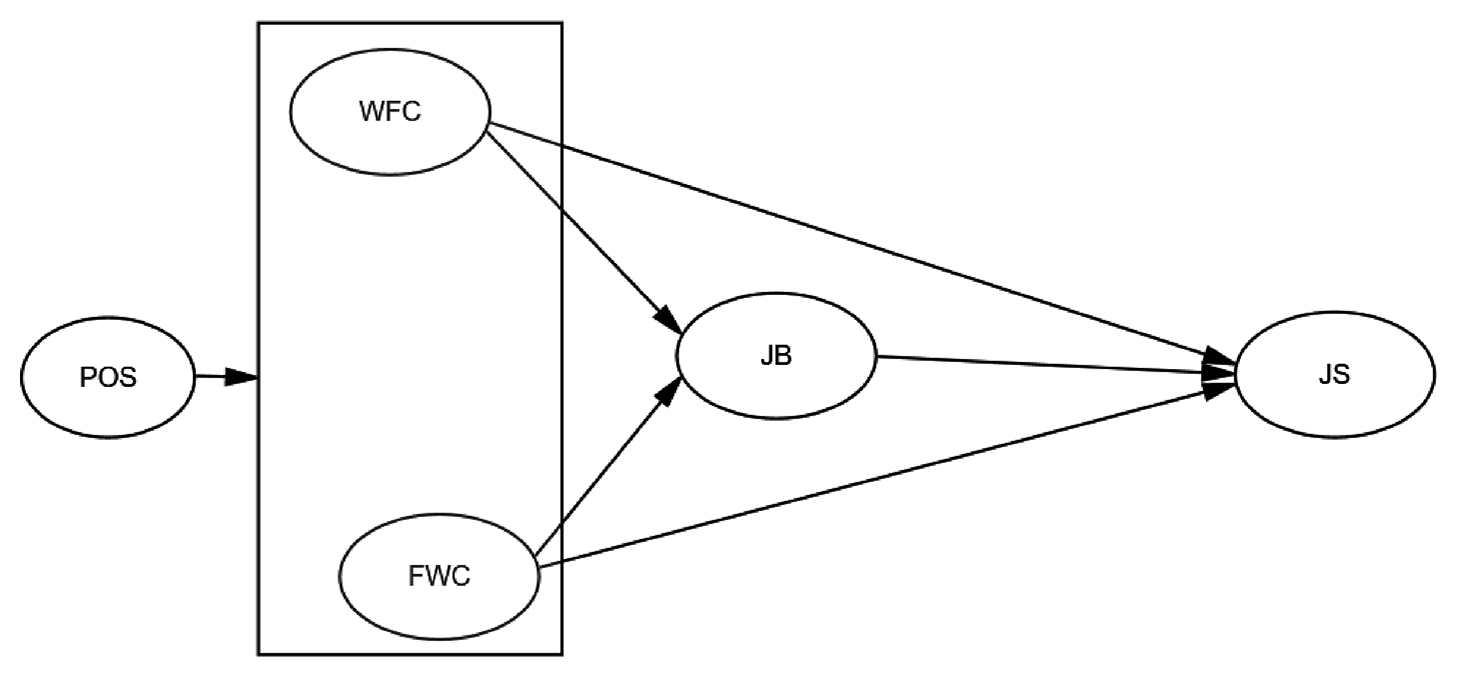
Theoretical framework model. WFC, Work Family Conflict; FWC, Family Work Conflict; JB, Job Burnout; JS, Job Satisfaction; POS, Perceived Organizational Support.

To sum up, this study constructed a mediation model with regulation (see Figure 1). The effects of bi-directional work–family conflict, job burnout and perceived organizational support on job satisfaction were investigated.

## 3. Method

### 3.1. Participants

This study used a convenient sampling method to conduct an online questionnaire survey among female university teachers in China. A total of 560 questionnaires were collected, and 527 valid questionnaires were retained, with an effective rate of 94.11%, excluding invalid questionnaires with short response time and unreasonable answers. Among them, there were 159 people under 30 years old, accounting for 30%, 289 people between 30 and 40 years old, accounting for 54.8%, 67 people between 41 and 50 years old and accounting for 12.7%, 12 people over 50 years old and accounting for 2.3%; Fertility: 89 (16.9%) have not given birth, 323 (61.3%) have one child, 113 (21.4%) have two children, 2 (0.4%) have more than three children, 153 (29%) with bachelor’s degree, 287 (54.5%) with master’s degree, 85 (16.1%) with doctor’s degree; There are 71 people with ungraded professional titles, accounting for 13.5%, 225 people with junior professional titles, accounting for 42.7%, 146 people with intermediate professional titles and accounting for 27.7%, and 85 people with senior professional titles and accounting for 16.1%; There are 127 people with less than 5 years of working life, accounting for 24.1%, 262 people with 5 to 10 years of working life, accounting for 49.7%, 108 people with 11 to 20 years of working time, accounting for 20.5%, and 30 people with more than 20 years working time, accounting for 5.7%.

### 3.2. Tools

#### 3.2.1. Work–family conflict scale

The Work–Family Conflict Scale (WFCS) made by Netemeyer et al. (1996) was adopted to assess the extent of bi-directional work–family conflict among female university teachers in China. The scale consists of 10 items, including two dimensions of work–family conflict and family–work conflict. Likert 5 points are used, ranging from “1” for “strongly disagree” to “5” for “strongly agree.” The higher the score is, the higher the work–family conflict will be. In this study, the Cronbach’s alpha coefficients of work–family conflict and family–work conflict were 0.882 and 0.870, respectively.

#### 3.2.2. Job satisfaction scale

The Minnesota satisfaction questionnaire (MSQ) made by Weiss et al. (1967) was adopted to assess the job satisfaction of female university teachers in China. The scale consists of 20 items, including three dimensions of intrinsic satisfaction, extrinsic satisfaction and general satisfaction. Likert 5 points are used, ranging from “1” for “very dissatisfied” to “5” for “very satisfied.” Higher scores indicate higher job satisfaction. The Cronbach’s alpha coefficient of the scale in this study was 0.953.

#### 3.2.3. Job burnout scale

The Job Burnout Inventory (Maslach Burnout Inventory, MBI) made by Maslach and Jackson (1981) was adopted to assess job burnout of female university teachers in China. The scale consists of 15 items, including three dimensions of emotional exhaustion, depersonalization and low sense of accomplishment. Likert 5 points are used, ranging from “1” for “strongly disagree” to “5” for “strongly agree.” Higher scores indicate higher job satisfaction. In this study, the Cronbach’s alpha coefficients of emotional exhaustion, depersonalization and low sense of accomplishment were 0.904, 0.866 and 0.821, respectively.

#### 3.2.4. Perceived organizational support scale

The perceived organizational support scale (Perceived Organizational Support Scale, POSS) made by Settoon et al. (1996) was adopted to assess the perceived organizational support of female university teachers in China. The scale contains seven items. Likert 5 points were used, ranging from “1” for “strongly disagree” to “5” for “strongly agree.” The higher the score is, the stronger the organizational support will be. The Cronbach’s alpha coefficient of the scale in this study was 0.897.

#### 3.2.5. Control variables

Studies have shown that demographic variables such as age, education level, income, job title, number of children in the family and so on have an impact on individual work–family conflict (Zacher and Winter, 2011; Kengatharan, 2015; Notten et al., 2017). Based on this, in order to ensure the validity of the study, this paper selects age, education level, job level, the number of children in the family, working years and so on as the control variables of this study.

### 3.3. Statistical processing

SPSS 25.0 and PROCESS statistical software were used for data entry and statistical analysis, and Pearson correlation analysis, Harman single factor test, mediating effect analysis and moderating effect analysis were used for statistical analysis.

## 4. Results

### 4.1. Common method bias test

In terms of statistical test, this study first uses Harman’s single-factor test method to conduct exploratory factor analysis on the main items involved in the study with the help of SPSS 25 software. The results of dimensionality reduction show that the variance explanation rate of the first unrotated factor is 39.031%, which is lower than the critical value of 50.00%, indicating that there is no significant common method bias in the research data.

### 4.2. Discriminant validity test

Confirmatory factor analysis was used to test the discriminant validity of five latent variables: work–family conflict, family–work conflict, job burnout, job satisfaction and perceived organizational support. It can be seen from Table 1 that, compared with other models, the 5-factor model has the best fitting effect, and each fitting index is at an acceptable level, indicating that the five main constructs in this paper have good discriminant validity.

**Table 1:** Confirmatory factor analysis.

Fit Index	χ^2^	df	χ2/df	RMSEA	CFI	IFI	TLI	SRMR
Five-factor model	3858.509	1264.000	3.053	0.062	0.858	0.858	0.851	0.071
Four-factor model	5086.836	1268.000	4.012	0.076	0.791	0.791	0.781	0.079
Three-factor model	5473.718	1271.000	4.307	0.079	0.770	0.770	0.760	0.082
Two-factor model	7043.245	1273.000	5.533	0.093	0.684	0.685	0.671	0.099
Single factor model	7389.941	1274.000	5.801	0.096	0.665	0.666	0.651	0.102

### 4.3. Descriptive statistical analysis and correlation analysis

The mean, standard deviation and correlation coefficient matrix of each variable in the paper are shown in Table 2. As can be seen from Table 2, the work–family conflict of female university teachers is negatively correlated with job satisfaction (*R* = −0.355), and family–work conflict is negatively correlated with job satisfaction (*R* = −0.446). At the same time, work–family conflict and family–work conflict are positively correlated with job burnout (*R* = 0.601). There was a significant negative correlation between job burnout and job satisfaction (*R* = −0.532). So far, Hypotheses 1 ~ 4 have been preliminarily confirmed.

**Table 2:** Mean, standard deviation and correlation coefficient of each variable.

	*M*	SD	AGE	BIRTH	EDU	TITLE	SL	WFC	FWC	POS	JB	JS
AGE	1.871	0.71	1									
BIRTH	2.055	0.637	0.369**	1								
EDU	1.879	0.673	0.317**	0.033	1							
TITLE	2.465	0.918	0.571**	0.304**	0.353**	1						
SL	2.078	0.818	0.731**	0.397**	0.128**	0.552**	1					
WFC	3.065	0.97	0.008	0.084	0.098*	0.101*	−0.049	1				
FWC	2.65	0.969	0.066	0.191**	0.188**	0.163**	0.041	0.636**	1			
POS	3.504	0.887	−0.246**	−0.162**	−0.211**	−0.254**	−0.263**	−0.283**	−0.359**	1		
JB	3.2	0.533	−0.031	0.064	0.078	0.057	−0.024	0.601**	0.520**	−0.464**	1	
JS	3.687	0.722	−0.198**	−0.179**	−0.216**	−0.230**	−0.216**	−0.355**	−0.446**	0.889**	−0.532**	1

^*^p < 0.05, ^**^p < 0.01.

### 4.4. Model hypothesis testing

#### 4.4.1. Main effect and mediating effect test

In this paper, hierarchical regression method is used to test the main effect and mediating effect of hypotheses 1 to 4, respectively. First of all, this paper examines the negative impact of work–family conflict and family–work conflict on job satisfaction of female university teachers. Secondly, this paper examines the negative impact of work–family conflict and family–work conflict on job burnout of female university teachers; Finally, we put work–family conflict, family–work conflict and job burnout into the regression equation at the same time, and verify that the effect of work–family conflict and family–work conflict on job satisfaction of female university teachers disappears or weakens when the effect of job burnout on job satisfaction is still significant. The test results are shown in Tables 3, 4.

**Table 3:** Regression analysis results of main effect, mediating effect and moderating effect of WFC.

	X → Y	X → Me	X,Me → Y	X,Me,Mo → Y	X,Mo → Me
	Model 1	Model 3	Model 5	Model 7	Model 9
Constant	5.292^**^	2.142^**^	6.725^**^	2.599^**^	2.030^**^
Age	0.047	−0.084^*^	−0.009	0.03	−0.070
Birth	−0.083	0.021	−0.069	−0.042	−0.006
Edu	−0.169^**^	0.036	−0.145^**^	−0.041	−0.004
Title	−0.027	−0.002	−0.029	0.003	−0.014
SL	−0.174^**^	0.048	−0.142^**^	−0.012	0.005
WFC	−0.253^**^	0.329^**^	−0.033	−0.198^**^	0.644^**^
JB			−0.669^**^	−0.141^**^	
POS				0.517^**^	0.131^*^
WFC*POS				0.044^**^	−0.103^**^
*R* ^2^	0.209	0.367	0.363	0.816	0.500
Adjusted *R*^2^	0.200	0.360	0.355	0.812	0.492
*F*	22.929**	50.219**	42.333**	254.442**	64.815**

**Table 4:** Regression analysis results of main effect, mediating effect and moderating effect of FWC.

	X → Y	X → Me	X,Me → Y	X,Me,Mo → Y	X,Mo → Me
	Model 2	Model 4	Model 6	Model 8	Model 10
Constant	5.119^**^	2.539^**^	6.596^**^	2.603^**^	2.879^**^
Age	0.014	−0.054	−0.018	0.018	−0.044
Birth	−0.03	−0.014	−0.038	−0.026	−0.027
Edu	−0.118^*^	−0.003	−0.120^**^	−0.029	−0.036
Title	−0.025	0.009	−0.02	0.006	−0.003
SL	−0.147^**^	0.004	−0.145^**^	−0.008	−0.043
FWC	−0.305^**^	0.289^**^	−0.136^**^	−0.247^**^	0.485^**^
JB			−0.582^**^	−0.128^**^	
POS				0.507^**^	0.004
FWC*POS				0.05^**^	−0.075^**^
*R* ^2^	0.251	0.275	0.385	0.822	0.400
Adjusted *R*^2^	0.243	0.267	0.377	0.818	0.390
*F*	*29.11* ^**^	*32.935* ^**^	*46.411* ^**^	*264.523* ^**^	*43.169* ^**^

Model 1 to model 2 showed that after controlling age, parenting situation, education, working years and professional title, work–family conflict and family–work conflict of female university teachers could significantly and negatively predict their job satisfaction (β = −0.253, *p* < 0.001; β = −0.305, *p* < 0.001), and hypotheses 1 and 2 were verified. Models 3 to 4 showed that work–family conflict and family–work conflict had significant negative predictive effects on job burnout after adding control variables (β = 0.329, *p* < 0.001; β = 0.289, *p* < 0.001). Model 5 showed that when both work–family conflict and job burnout were included in the equation, the negative prediction effect of work–family conflict on job satisfaction of female university teachers was not significant (β = −0.033,n.s.). However, job burnout was still a significant predictor of job satisfaction (β = −0.669, *p* < 0.001). Model 6 showed that when both family–work conflict and job burnout were included in the equation, the prediction effect of family–work conflict on job satisfaction was still significant (β = −0.136, *p* < 0.001), while the prediction effect of job burnout on job satisfaction was still significant (β = −0.582, *p* < 0.001). From Model 1 to Model 6, it can be seen that job burnout plays a partial mediating role in the process of work–family conflict and family–work conflict affecting job satisfaction of female university teachers. Thus, assumptions 3 and 4 are verified. In order to further verify the mediating effect of job burnout, this paper uses Process plug-in and Bootstrap method to repeatedly extract 1,000 times. The results show that the mediating effect value of job burnout between work–family conflict and job satisfaction is −0.220, and the 95% confidence interval is −0.359, −0.234. The mediating effect of job burnout between family–work conflict and job satisfaction was −0.168, and the 95% confidence interval was −0.286, −0.171. Hypothesis 3 and 4 were verified again.

#### 4.4.2. Moderating effect test

This paper uses hierarchical regression method to test the moderating effect of perceived organizational support on work–family conflict, family–work conflict, job burnout and job satisfaction. Before regression analysis, the independent variables and the adjusted variables were centralized, and then the control variables were put into the regression equation, and then work–family conflict, family–work conflict and perceived organizational support were put into the regression equation at the same time. Finally, the interaction terms of work–family conflict and perceived organizational support, as well as the interaction terms of family–work conflict and perceived organization support were put into regression equation. See Tables 3, 4 for the effect.

Model 7 showed that after controlling the main effects of work–family conflict, job burnout and perceived organizational support, the interaction of work–family conflict and perceived organizational support had a significant impact on job satisfaction (β = −0.198, *p* < 0.001), indicating that perceived organizational support had a moderating effect on the relationship between work–family conflict and job satisfaction, and hypothesis 5a was preliminarily verified; Model 8 showed that after controlling the main effects of family–work conflict, job burnout and perceived organizational support, the interaction between family–work conflict and perceived organizational support had a significant impact on job satisfaction (β = −0.247, *p* < 0.001), indicating that perceived organizational support had a moderating effect on the relationship between family–work conflict and job satisfaction, and hypothesis 5b was preliminarily verified; Model 9 shows that, after controlling the main effects of work–family conflict and perceived organizational support, the interaction of work–family conflict and perceived organizational support has a significant effect on job burnout (β = 0.644, *p* < 0.001), indicating that perceived organizational support has a moderating effect on the relationship between work–family conflicts and job burnout. Hypothesis 5c has been preliminarily verified; Model 10 showed that, after controlling the main effects of family–work conflict and perceived organizational support, the interaction of family–work conflict and perceived organizational support had a significant effect on job burnout (β = 0.485, *p* < 0.001), indicating that perceived organizational support had a moderating effect on the relationship between family-work conflicts and job burnout, and hypothesis 5d was preliminarily verified.

To further measure the moderating effect of perceived organizational support, based on suggestion of Curran et al. (1996), Take the next standard deviation of perceived organizational support to draw the moderating effect map, and make a simple slope analysis, then use Jonson-Neyman technique to detect the statistically significant interval of the moderating effect, and form a simple slope change trajectory.

**Figure 2:**
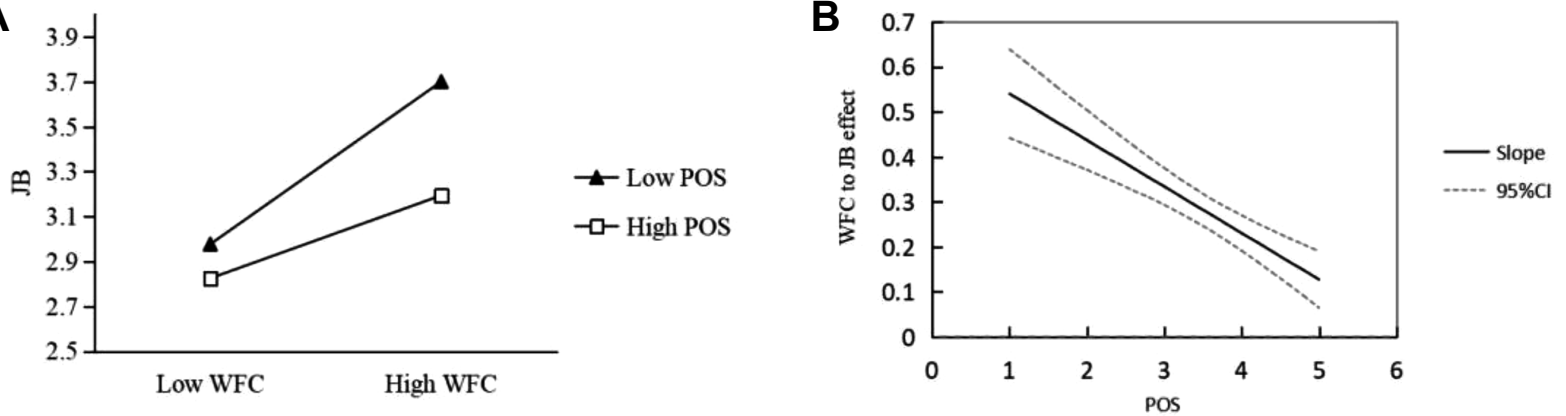
Moderating effect of POS on the relationship between WFC and JB **A**: Simple slope test of moderating effect **B**: Johnson-Neyman analysis of moderating effect.

As shown in Figure 2, the positive moderating effect of perceived organizational support on job burnout in the process of work–family conflict: for female university teachers with high perceived organizational support (1 standard deviation higher than the average), the impact of work–family conflict on job burnout is relatively weak, with an effect value of 0.190; For female university teachers with low perceived organizational support (1 standard deviation below the mean), the impact of work–family conflict on job burnout is relatively more obvious, and the effect value is 0.373.

**Figure 3:**
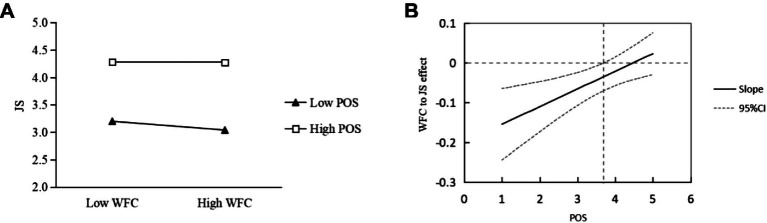
Moderating effect of POS on the relationship between WFC and JS **A**: Simple slope test of moderating effect **B**: Johnson-Neyman analysis of moderating effect.

As shown in Figure 3, the negative moderating effect of perceived organizational support in the process of work–family conflict affecting job satisfaction: for female university teachers with high perceived organizational support (1 standard deviation higher than the average), the impact of work–family conflict on job satisfaction is relatively weak, with an effect value of −0.04; For female teachers with low perceived organizational support (1 standard deviation below the mean), the impact of work–family conflict on job satisfaction is relatively more obvious, and the effect value is −0.082. When the original score of perceived organizational support is higher than 3.683, the confidence interval includes 0, and the impact of work–family conflict on job satisfaction is not significant.

**Figure 4:**
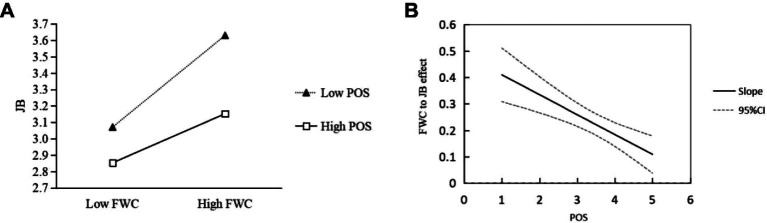
Moderating effect of POS on the relationship between FWC and JB **A**: Simple slope test of moderating effect **B**: Johnson-Neyman analysis of moderating effect.

As shown in Figure 4, the positive moderating effect of perceived organizational support on job burnout in the process of family–work conflict: for female university teachers with high perceived organizational support (1 standard deviation higher than the average), the effect of family–work conflict on job burnout is relatively weak, with an effect value of 0.155; For female university teachers with low perceived organizational support (1 standard deviation below the mean), the impact of family–work conflict on job burnout is relatively more obvious, and the effect value is 0.288.

**Figure 5:**
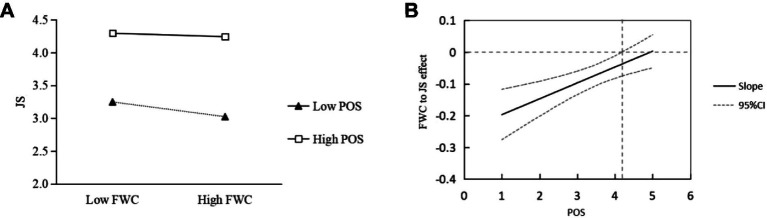
Moderating effect of POS on the relationship between FWC and JS **A**: Simple slope test of moderating effect **B**: Johnson-Neyman analysis of moderating effect.

As shown in Figure 5, the negative moderating effect of perceived organizational support on the impact of family–work conflict on job satisfaction: for female university teachers with high perceived organizational support (1 standard deviation higher than the average), the impact of family–work conflict on job satisfaction is relatively weak, with an effect value of −0.020; For female university teachers with low perceived organizational support (1 standard deviation below the mean), the impact of family–work conflict on job satisfaction is relatively more obvious, and the effect value is −0.037. When the original score of perceived organizational support is higher than 4.189, the confidence interval includes 0, and the impact of family–work conflict on job satisfaction is not significant.

#### 4.4.3. Moderated mediating effect test

In order to ensure the robustness of the test results, this paper uses the regulatory mediation index to test the mediated mediation. It can be seen from Table 5 that the mediating index of the two paths is significant in the 95% confidence interval. Therefore, it can be concluded that perceived organizational support has a moderating effect on the impact of work–family conflict and family–work conflict on job satisfaction.

**Table 5:** Moderated mediating index.

Independent variable	Regulating variable	Mediating variable	Index	BootSE	BootLLCI	BootULCI
WFC	POS	JB	0.015	0.005	0.005	0.025
FWC	POS	JB	0.010	0.004	0.002	0.018

## 5. Discussion

The empirical results show that both work–family conflicts and family-work conflicts have significantly negative effects on the job satisfaction of female university teachers; job burnout plays a part-mediating role in the impact of work–family conflicts and family-work conflicts on the job satisfaction of female university teachers; and perceived organizational support positively moderates the effect of work–family conflict and family–work conflict on job satisfaction of female university teachers through job burnout.

### 5.1. The relationship between B-directional work–family conflict and job satisfaction of female university teachers

Judging from the results of previous studies, the conclusion of the relationship between work–family conflict and job satisfaction is very different (Allen et al., 2012). Most studies have concluded that both WFC and FWC are significantly negatively correlated with job satisfaction, but the degree of influence in this study is relatively small. In the Chinese context, there is a widespread and strong work-first social norm, a stronger tolerance for work interfering with the family, and it is a traditional virtue to work hard at the expense of one’s family (Gao, 2021). Even if there is conflict between work and family, it is considered normal and will not reduce job satisfaction. When Chinese employees have conflicts between work and family, the family will generally give way to work. People feel more about the impact of work on family life and less about the interference of family on work (Luo and Shen, 2017). However, in this study, compared with work–family conflict (effect value −0.198), family–work conflict (−0.247) has a greater impact on job satisfaction, which may be due to the higher education, higher income, as well as higher expectations of the sample participants.

### 5.2. Mediating effect of job burnout

This study found that job burnout partially mediated the relationship between bi-directional work–family conflict and job satisfaction. The relationship between job burnout and job satisfaction has been widely verified. The teaching profession is a profession with great pressure, and the burnout of teachers gradually increases with the increase of pressure (García-Carmona et al., 2019). According to the COR, female university teachers will consume a lot of resources when they are faced with work–family conflicts. Since the stock resources are limited, individuals feel threatened by resource depletion and will spend resources to seek replenishment from elsewhere, which invisibly causes individuals to fall into the spiral of resource loss, resulting in job burnout (Hobfoll, 2001). In addition, job burnout significantly affects job satisfaction, which is also consistent with the existing research, that is, the higher the job burnout, the lower the job satisfaction, and job satisfaction has a significant negative predictive effect on job burnout (Gómez-García et al., 2020). For female university teachers, work–family conflict mainly indirectly affects job satisfaction through job burnout. Compared with other practitioners, female university teachers are influenced by traditional social gender concepts and limited personal energy, and bear higher work–family conflict, which will lead to higher levels of job burnout and physical and mental health crisis (García-Carmona et al., 2019). Therefore, helping female university teachers to reduce the level of job burnout is helpful to improve their job satisfaction and physical and mental health.

### 5.3. Moderating effect of perceived organizational support

Perceived organizational support plays a moderating role in the relationship among bi-directional work–family conflict, job burnout and job satisfaction of female university teachers, and its role in the mediation process occurs in the first half of the path. When the perceived organizational support of female university teachers is higher, the level of job burnout caused by bi-directional work–family conflict will be reduced, and the impact on job satisfaction also declined (Lee and Peccei, 2007). Because the increase of perceived organizational support helps to improve the relationship between employees and the organization, increase the emotional commitment and job satisfaction of employees to the organization, and improve job performance (Gao, 2021). In collectivist China, there is a mutual spillover between the family field and the work field. When employees feel upset and annoyed about family life, their superiors or colleagues may show their understanding and support (Luo and Shen, 2017). In the study of university teachers, it is also believed that good perceived organizational support can effectively reduce teachers’ job burnout and improve teachers’ job satisfaction. The results of this study are also consistent with COR. For female university teachers, high organizational support is an important external job resource from individuals. Job resources can start the incentive process, promote individuals to have a higher level of job engagement, and then have a positive effect on their job performance and mental health, thereby enhancing their job satisfaction; In addition, work resources have a buffer effect, which can compensate for the negative impact of excessive work demands on individual health and well-being so as to reduce job burnout (Hobfoll, 2001).

The relevant departments of the university can consult the female teachers themselves, inquire about the relevant policy support they perceive, find out the problems related to policy implementation and solve them in time, enhance the sense of organizational support of teachers, so as to ease the various types of conflicts between work and family, improve their job satisfaction, mobilize their enthusiasm for work, and contribute more to the cause of education (Gao, 2021). At the same time, the improvement of working ability can help female university teachers resist pressure and depression, and help female teachers solve the difficulties caused by work pressure (Khamisa et al., 2015). Therefore, universities should actively respond to the requirements of the education sector, regularly require female teachers to participate in training, promote female teachers to achieve personal recharge, and improve their teaching and research capabilities (Lin, 2022). Moreover, universities should hold regular group outings, such as teachers’ parties and mountaineering tours, encourage female teachers to actively participate in activities, enhance mutual understanding between female teachers and colleagues, and help female teachers relieve pressure and relax their body and mind (Li et al., 2021). So as to improve the level of perceived organizational support of female university teachers, alleviate their work–family conflict, and enhance job performance and job satisfaction.

### 5.4. Limitations and future directions

First of all, due to the limitations of research conditions, we do not use dynamic research methods, but use static research methods. However, teachers’ job satisfaction is not fixed, and job burnout changes over time. In future research, a combination of static and dynamic survey methods, such as daily diary method, should be considered as far as possible to test the hypothesis more effectively. Second, this paper does not consider the mediating factors that hinder stress. Next time, we will explore the influence of other types of organizational environments on job burnout and further explore the influence of two-dimensional stressors in different directions. Thirdly, this paper only selects some outcome variables in the field of work to study, but does not include the outcome variables in the field of family, such as life satisfaction, marriage satisfaction and family satisfaction. Future research can examine whether the relationship between work and family will affect the quality of life outside work of female teachers in China, Such as physical health (whether there are restrictions on various physiological functions and activities, whether rest and sleep are normal, etc.), mental health (intelligence, emotion, stress stimulation, etc.), social health (social interaction and social activities, family relations, social status, etc), mental health (understanding of the value of life, religious beliefs and spiritual culture, etc).

## Data availability statement

The raw data supporting the conclusions of this article will be made available by the authors, without undue reservation.

## Ethics statement

Informed consent was obtained from all subjects involved in the study. Ethical review and approval was not required for the study on human participants in accordance with the local legislation and institutional requirements. Written informed consent from the participants was not required to participate in this study in accordance with the national Legislation and the institutional requirements.

## Author contributions

All authors listed have made a substantial, direct, and intellectual contribution to the work and approved it for publication.

## Conflict of interest

The authors declare that the research was conducted in the absence of any commercial or financial relationships that could be construed as a potential conflict of interest.

## Publisher’s note

All claims expressed in this article are solely those of the authors and do not necessarily represent those of their affiliated organizations, or those of the publisher, the editors and the reviewers. Any product that may be evaluated in this article, or claim that may be made by its manufacturer, is not guaranteed or endorsed by the publisher.
